# Fracture of the Jaw

**Published:** 1893-12

**Authors:** R. Y. Henley

**Affiliations:** Danville, Va.


ARTICLE IX.

### FRACTURE OF THE JAW.

BY DR. R. Y. HENLEY, DANVILLE, VA.

Read before the Virginia State Dental Society, August 8th, '93.

Fracture of the jaw is usually the result of violence, though fracture of the neck of the bone has in a few instances in very old persons resulted from a violent fit of coughing.

Blows on the jaw from falling, fighting or the kick of a horse are some of the most common causes.

The unskilful use of the key or forceps in the extraction of teeth has produced complete fracture of the jaw, which generally from that cause occurs in the ramus of the inferior maxilla. Gunshot wounds of the face often produce most terrible injuries of the jaw, by splintering or removing large portions of it. Fractures of the inferior maxilla are remarkable from the fact that they are so frequently compound, while the skin is rarely ruptured except in gunshot wounds. The fibrous tissue of the gums, being very inelastic, tears easily when the bone is broken across, which enables the saliva and air to come in contact with the fractured surfaces.

Fractures of the ramus, the condyle or coronoid process are, however, too deeply seated for the injury to effect the membrane of the mouth. Fractures may occur at various points in the lower jaw, but the body of the bone is the portion most frequently injured—the ramus from its position and coverings being much less liable to injury except from extreme violence.

The coronoid process is sometimes broken off obliquely, and the neck of the jaw is often broken on one or both sides of the bone in cases of extreme violence.

In the body of the jaw the fracture occurs most frequently in the region of the canine tooth, on account of the greater depth of the socket and the consequent weakness of the bone at that point. It may, however, occur at any other point.

The line of fracture, except at the symphysis, is usually oblique. In double fractures of the body of the jaw, one being on each side of the median line, the displacement is necessarily greater, as the muscles attached to the chin have a tendency to draw the central loose piece downward and backward toward the hyoid bone, while both latteral pieces are drawn forward and outward. Reduction of a fracture of the neck of the jaw, where complete displacement is produced, can only be accomplished by acting upon the condyle and the jaw at the same time. The finger carried far back in the mouth should throw the condyle out, while the jaw is brought into its proper relation with the other hand. The fragments must then be pressed firmly together and against the glenoid cavity by means of a bandage.

Fracture of the coronoid process rarely occurs, and it is claimed by some when it does occur osseous union never takes place, but I am free to say that the claim will not hold good except in a few cases of very old persons.

Considerable inflammation frequently follows fracture, even of a simple nature, especially if it has been neglected for some hours. The face becomes swollen and tissues beneath the chin infiltrated with serum, which is sometimes converted into pus, giving rise to troublesome abscesses.

Wounds of the face rarely accompany fracture of the jaw except, as I have stated, in gunshot wounds, and when they do occur the wounds are treated on ordinary principles, and are of little consequence so far as the fracture is concerned, except that they interfere with the application of the necessary retentive apparatus or bandage. Hemorrhage, beyond that caused by the laceration of the gum, is rarely met with. The elasticity of the inferior dental artery enables it to stretch sufficiently, in a majority of cases, to avoid rupture. Should the artery be ruptured the hemorrhage can usually be controlled by digital compression of the caroted artery for two or three hours.

Dislocation and fracture of the teeth is frequently met with, the former being the direct result of a blow, or the consequence of a fracture runniug through the socket, and the latter the result of direct violence. In the region of the molass it is often caused by the indirect force of the neighboring teeth, and frequently by the teeth being driven against those of the upper jaw. Where the fracture passes through the socket the tooth may fall between the edges of the bone, and thus seriously interfere with the proper reduction of the fracture. This fact should always be borne in mind when a tooth is missing and difficulty is experienced in settling the fracture.

In fracture of the lower jaw in children (a very rare accident), where it happens to involve the cavity in which a permanent tooth is being developed, exfoliation of the tooth, with a portion of the alveolus, is almost certain to ensue. Paralysis and neuralgia from injury to the inferior dental nerve may be the immediate result of the accident, or be caused at a later period by pressure. In a majority of cases the nerve sustains little or no injury, which is due to the fact that a greater number of fractures occur between the symphysis and the inferior dental foramen through which the nerve passes.

Abscess frequently occurs in severe injuries of the jaw, the matter pointing below the bone. This condition is as often caused by undue pressure of the retentive apparatus as by the injury. A certain amount of pus usually finds its way into the mouth through the lacerated gums in all cases of severe fracture, but the exit is usually sufficient to prevent the occurrence of abscess within the mouth. In neglected cases of fracture the abscess may be connected with necrosis, and may point at some distance down the neck, and the fistulous opening remain for several months or even yeare.

Salivary fistula may result from a compound fracture of the inferior maxilla or from an abscess bursting externally in case of a simple fracture—the treatment of which is the same as that arising from other causes, such as necrosis, etc.

Necrosis of the alveolar process frequently follows fracture, to a limited extent, without producing any permanent deformity; but it sometimes effects the body of the bone to such an extent that its removal is necessary and very great deformity follows.

Dislocation of the jaw sometimes accompanies fracture, but is exceedingly rare, from the fact that fracture tends to prevent it. Where the displacement of the fragments of the bone has been great it is sometimes impossible to keep them in proper position, and the result is an irregular union of the bone, which may interfere with its functions in after life. This condition is more liable to occur in cases of double fracture, where the central portion of the jaw is very much displaced by the muscles attached to it.

Fractures of the lower maxilla usually unite with great rapidity and certainty, in spite of the difficulty of keeping the fragments in proper position. Non-union may be simply the result of neglect of treatment, and union may take place readily as soon as the parts are placed under favorable circumstances. Necrosis at the point of fracture is one of the most prominent causes of non-union, and for that reason we find that fractures from gunshot wounds are frequently difficult to unite. The amount of inconvenience from an ununited fracture varies according to the position of the false joint. In the ramus it seems to give very little, if any, inconvenience—the new joint performing the functions of the temporo-maxillary articulation. When a false joint occurs in the body of the bone it often interferes with mastication very seriously.

The treatment of fracture of the lower jaw after the reduction of any displacement is generally of a simple nature, but cases sometimes arise in which the most carefully adapted mechanical appliances fail to effect a good union. The apparatus employed to hold the fractured parts in position may be divided into two classes, viz.: those which are applied externally, and those which are applied in the oral cavity. It is sometimes necessary to combine the two methods in extreme cases. The simplest form of external apparatus consists of the ordinary four-tailed bandage. When the condyle is displaced by the action of the pterygoidus externus reduction must be effected by drawing the jaw horizontally forward, and at the same time pushing the condyle outward with the finger introduced far back into the mouth. Reduction being accomplished, the jaw must be pressed upward and backward to fix the condyle in the glenoid cavity, after which a bandage may be applied. When delay is due to superficial necrosis, time for exfoliation to take place is all that is required. When the necrosis is extensive, or the loss of substance great, it is not desirable to produce union between the fragments, as an unsightly deformity will be induced, which can be avoided by the use of apparatus to retain the parts in their normal positions,

Fracture of the superior maxilla are not nearly so common as those of the inferior, but their results are more serious, from the fact that the patient has to undergo greater violence.

Fractures of the upper jaw may be produced by a fall on the face and striking on the molar bone, which often produces vertical fracture through the orbitar process of the superior maxilla. Direct blows on the bone are the most common causes of fracture. Fracture extending into the antrum often gives rise to suppuration in that cavity, but it is not a necessary consequence.

The nasal process of the superior maxilla has been fractured by blows which have also driven in the nasal bone, and in these cases emphysema of the cellular tissue of the face is not uncommon, which is best checked by the application of collodion. A complication of this form of fracture, which has been met with, is permanent obstruction of the nasal duct, leading to subsequent troublesome epiphora.

Separation of the maxilla in the median suture has been seen in cases of fatal injury to the face. Recovery from this condition is unusual. The teeth of the upper jaw may be broken or dislocated as in fracture of the lower jaw; but, if they are merely loosened, should never be removed, as they will become firmly attached.

Splintering of the bone is much more common in the upper than in the lower jaw. Hemorrhage is much more frequent and copious in fractures of the upper than those of the lower jaw, because of the great vascularity of the parts. Hemorrhage frequently comes from rupture of the internal maxillary artery, and may be immediately fatal. Secondary hemorrage is a frequent occurrence.

Injury to the infra-orbital nerve and its branches often ensues in cases of severe fracture and comminution of the superior maxilla, and consequent numbness or modification of sensation will be the result. Serious brain symptoms may ensue when the fracture runs back to the sphenoid bone, as the fissure may extend to the cranium. This trouble is especially liable to occur when the whole of the septum narium is driven back with the jaw.

Fractures of the upper jaw require but little treatment as compared with the lower, for the reason that the part is usually so much more fixed than that of the lower jaw, and consequently the less difficulty in keeping the fragments in position.

Hemorrhage, which is sometimes copious, should be arrested by cold, the application of styptics, and, as a last resort, the actual cautery.

When, as is frequently the case, the soft tissues of the face are lacerated, and the hemorrhage arises from them, the bleeding vessels should be secured with ligatures in the ordinary manner. It is not best to remove the fragments of the upper jaw bone, for the reason that the parts are vascular, and will nearly always unite readily.—*Southern Dental Journal.*

---

